# Characterization of a new cell line from ornamental fish *Amphiprion ocellaris* (Cuvier, 1830) and its susceptibility to nervous necrosis virus

**DOI:** 10.1038/s41598-020-76807-7

**Published:** 2020-11-18

**Authors:** B. S. Yashwanth, Mukunda Goswami, Rajendran Kooloth Valappil, Dimpal Thakuria, Aparna Chaudhari

**Affiliations:** 1 Fish Genetics and Biotechnology Division, ICAR-Central Institute of Fisheries Education, Mumbai, 400061 India; 2 Aquatic Environment and Health Management Division, ICAR-Central Institute of Fisheries Education, Mumbai, 400061 India; 3 Genetics and Biotechnology Section, ICAR-Directorate of Coldwater Fisheries Research, Bhimtal, 263136 India

**Keywords:** Developmental biology, Cell growth, Cell proliferation

## Abstract

*Amphiprion ocellaris* (ocellaris clownfish) is one of the most commercially important marine ornamental fish. A cell line designated as OCF was developed for the first time from the caudal fin of this fish species. The cell line was maintained in Leibovitz’s—15 medium supplemented with 15% FBS (Fetal Bovine Serum) and was successfully subcultured up to 34 passages. The cell line was authenticated by sequencing mitochondrial cytochrome C oxidase subunit I (COI) and 16S rRNA genes. The growth rate of the OCF cell line was maximum in medium containing 20% FBS and 1% of 0.2 M NaCl at 28 °C. Chromosome analysis revealed 48 diploid chromosomes. The OCF cell line was transfected with the pMaxGFP plasmid vector with 7% efficiency and GFP expression was observed. The OCF cell line was used for testing nervous necrosis virus (NNV) susceptibility. Cytopathic effect (CPE) was observed in terms of plaque formation after virus inoculation. Nested PCR confirmed the susceptibility of the OCF cell line to NNV. The cell line was successfully cryopreserved by a slow freezing procedure at − 80 °C with a revival efficiency of 70–75%. The study revealed that the OCF cell line would be useful for virological studies. In addition, the cell line would play an important role as an in vitro tool for carrying out toxicological and biotechnological studies.

## Introduction

Cell lines have been used as vital in vitro tools for performing various studies in life science including their best applications for various studies i.e. studying virology, environmental toxicology, cytobiology, oncology, drug screening and development, gene expression studies, genetics and genomics^1,2^. Fish cells have an advantage over mammalian or avian cells with minimal maintenance requirement, replicate within a broad range of incubation temperatures, and a flexible culture program^3^. These distinctive features of fish cells make them useful tools for a variety of biological requisitions in life science. The first permanent cell line from fish was developed from the gonads of Rainbow trout, *Salmo gairdneri* (RTG2)^4^. Since then, many fish cell lines have been established using a broad variety of tissues representing marine and freshwater fish. Bairoch has provided more updated details enlisting 883 fish cell lines worldwide in Cellulosaurus; a knowledge resource on cell lines^5^. Characterization of the cultured cells is one of the important parameters for cell line authentication, i.e., to confirm the species of origin and biology of the cultured cell line.

Almeida et al*.* reported the standard methods for authentication of cell lines such as cytochrome c oxidase subunit1 (COI) barcode, karyotyping, short tandem repeat (STR) profiling and single nucleotide polymorphisms (SNP) profiling^6^. Some other properties of cell lines including plating efficiency, which provides the proliferation capacity of the cell line, transfection efficiency of the foreign DNA for the gene expression studies, viability assay after cryopreservation. Cryopreservation of cultured fish cells more often relies on very simple and facile protocols using cryoprotectant DMSO (Dimethyl Sulfoxide). The DMSO is added to the cultured cell suspension in the medium, and short-term cryopreservation is carried out by keeping the cells in − 80 °C freezer^7,8^.

*Amphiprion ocellaris* is a marine ornamental fish belong to the Family *Pomacentridae* and subfamily *Amphiprioninae*. It is recognized as the third most exported ornamental fish. *A. ocellaris* is naturally distributed along Eastern Indian Ocean and Indo-West Pacific Ocean including the Andaman and Nicobar Islands, Philippines, Thailand, Malaysia, Singapore, Indonesia, North-west Australia, Taiwan, and Ryukyu Islands^9,10^. The catch of the clownfish has reduced dramatically in the last few years because of over-exploitation in response to its increasing demand, popularity, and worsening of its natural habitats. These scrutinize have led to the captive breeding of these marine ornamental fish, for conservation as well as commercial purposes^11,12^. Further, clownfish are susceptible to lymphocystis disease virus (LCDV) and cause mass mortality^13,14^. However, to undertake in vitro studies on the viruses infecting the species, a suitable cell line is not available. In this background, a cell line was developed for the first time using the caudal fin of marine ornamental fish, *Amphiprion ocellaris*. The cell line developed was further examined for its susceptibility to nervous necrosis virus (NNV). The NNV is a single-stranded RNA virus that comes under the family *Nodaviridae*. Even though both NNV and LCDV completely different viruses concerning virus biology and disease manifestation in fish hosts but these are the most common virus infecting the marine fish. Scherbatskoy et al., reported experienced periodic mortality in ocellaris clown fish due to picorna-like viruses which are genetically characterized as betanodavirus^58^. The mass mortality was observed in hatchery-reared larvae of clownfish due to NNV or betanodavirus^59^.

## Materials and methods

### Ethical committee

Institutional Animal Ethics Committee (IAEC), ICAR-Central Institute of Fisheries Education, Mumbai—61. For fish, they are not issuing any permit Id, as this work has been approved by the Institutional Research Committee (IRC) and Board of Studies (BOS).

We confirm the statement that:

- All experimental protocols were approved by the Institutional Animal Ethics Committee of ICAR-Central Institute of Fisheries Education, Mumbai, India.
- All methods were carried out in accordance with relevant guidelines and regulations approved by the institute and advisory committee, Fish Genetics and Biotechnology Division, ICAR-CIFE, Mumbai, India.

### Ethical treatment of animals or ethical approval

As for the Indian rules, there is no strict provision for permit Id for fish. However, the research work has been approved by IRC/BOS which is normally practiced in our institute.

All applicable international, national, and/or institutional guidelines for the care and use of animals were followed and that is approved by the institute.

### Primary cell culture

Normal and healthy live specimens of *A. ocellaris* (body weight: 1.5 ± 0.25 g; total length: 3.6 cm) originally collected from Reef Aquaria, Mumbai in live condition were transported to the Wet Laboratory of ICAR-Central Institute of Fisheries Education, Mumbai, Maharashtra and maintained in an aquarium with seawater. The donor fish were kept in well-aerated sterile seawater without feeding for 24 to 36 h. The fish was exposed to rapid hypothermic shock in an ice-chilled bath for 1–2 min. The caudal fin, eye, heart, gill, liver, and skin tissues were taken out aseptically and washed with 1 mL PBS containing 500 µg/mL streptomycin and 500 IU/mL penicillin and 2.5 μg/mL fungizone. The tissues were then minced into small pieces using a pair of sterile surgical scissors. The explants of 1 mm^3^ size were prepared and washed thrice with PBS (Thermo Fisher Scientific) containing antibiotics. The minced explants were then seeded into 25 cm^2^ cell culture flasks. The adherence of explants was accomplished by the addition of 0.2 mL of heat-inactivated Fetal Bovine Serum (FBS) (Gibco); then the flasks were incubated at 28 °C and allowed to attach properly to the surface of the flask by keeping the flask in the incubator. After 18–24 h, L-15 (Leibovitz) (HiMedia) medium supplemented with 20% FBS was added gently. The medium was changed after 3–5 days. The radiations of cells from the caudal fin showed faster compared to other tissues and it was used for the cell line development.

### Subculture and maintenance

Once the cells reached the confluency of 80–90%, the old medium was removed followed by rinsing the monolayer of cells with PBS. The cells were detached by trypsinization with 1–2 mL of trypsin–EDTA (0.25%) until the cells got completely detached from the flask surface. The detached cells were resuspended in 5 mL of L-15 fresh growth medium supplemented with 20% FBS and seeded in 25 cm^2^ cell culture flasks from the second passage onwards a split ratio of 1:2 was maintained for subsequent passages. In the initial subcultures, 50% of the culture medium was replaced with a fresh medium. The cells were incubated at optimum pH 7.4 and temperature 28 °C. The cultures were monitored daily and subcultured upon reaching 80% confluency^34,43^.

### Growth studies

#### Serum concentration(s)

Cells at a concentration of 2 × 10^4^ were seeded in 25 cm^2^ cell culture flasks having L-15 containing 20% serum and incubated overnight at 28 °C. The succeeding day, the medium was removed and thoroughly washed with PBS, and fresh culture medium containing different concentrations of FBS such as 5, 10, 15 and 20% FBS was added to the different cell culture flask and incubated at 28 °C and observed for seven days^15^. At every 24 h intervals, the relative number of viable and live cells in triplicate flasks in each set was estimated using a hemocytometer under an inverted microscope.

#### Salt concentration(s)

Cells were seeded at a density of 2 × 10^4^ in 25 cm^2^ cell culture flasks having L-15 medium supplemented with 20% FBS and incubated at 28 °C overnight. From the next day onwards, the medium was removed completely, and the cells were washed once with PBS, and the fresh culture media with 20% FBS supplemented with different 0.2 M NaCl concentrations such as 0.5%, 1%, 1.5%, and 2% was added to the different flasks. The culture flasks were incubated at 28 °C and observed for 7 days^16^. Every 24 h post-subculture, the relative number of viable cells in each set was estimated microscopically using a hemocytometer.

#### Temperature(s)

To assess the optimum growth response at different temperature ranges, cells were seeded at a density of 2 × 10^4^ in 25 cm^2^ cell culture flasks having L-15 containing 20% serum and incubated for overnight at 28 °C^15^. Next day onwards the cells were incubated at different temperature ranges such as 20, 24, 28, and 32 °C in 25 cm^2^ cell culture flasks at an initial concentration of 2 × 10^4^ cells/mL in culture medium having 20% serum. Every 24 h post-subculture, the relative number of live cells in each set was estimated using a hemocytometer.

### Plating efficiency

The plating efficiency of the cells was estimated according to the methods described by Ham and Puck^17^. Cells were trypsinized, counted, and diluted and seeded at 50, 100, 200, 500, 1000, and 2000 number of cells per well in a 6-well plate, in 1 mL L-15 medium containing 20% FBS and incubated at 28 °C. The culture medium was replaced three times with fresh medium in a week for 14 days. The cultures were rinsed, fixed with anhydrous methanol, and stained with Giemsa, and then colonies were counted.

Plating efficiency was calculated using the formula:

$${{P}}lating\, efficiency=\frac{No. \,of \,colonies}{No. \,of \,cells \,seeded}\times 100$$

### Measurement of cell doubling time

Cell doubling time (CDT) is the time interval required for a cell population to double in the middle of the logarithmic phase of cell growth. CDT was calculated using the following formula.

Cells/population doubling time (DT).

$$Cell \,doubling \,time=Incubation \,time \times ln\frac{cell\, number \,at\, the\, end \,of \,the\, incubation\, time }{cell\, number \,at \,the \,beginning\, of\, the\, incubation\, time}$$

### Authentication of cell line using molecular markers

#### Amplification of COI and 16S rRNA genes and sequencing

Genomic DNA from the Ocellaris clownfish fin (OCF) cell line was isolated following Sambrook^18^. For amplification of the mitochondrial COI gene, the universal pair of primers FishF1 and FishR1^19^ were used, and for amplification of 16S rRNA gene, 16sf1F140 and 16sf1R1524 primers (Xcelris) were used^20^. The details of primers, master mix preparation, and thermal regime of COI and 16S rRNA are mentioned in Tables 1, 2, 3 and 4. PCR products were visualized on 1.0% agarose gel by staining with ethidium bromide (EtBr) and documented using a gel documentation system (OmegaLum G, Aplegen, USA). The amplified products of both COI and 16S rRNA were sequenced and the sequences were analyzed using BLASTn.

**Table 1:** List of primers used for the amplification of mitochondrial genes COI and 16S rRNA.

Mitochondrial region	Primer Name	Primer sequence (5′-3′)	Length (bp)	References
COI	Fish F1	TCAACCAACCACAAAGACATTGGCAC	26	Ward et al*.*^19^
	FishR1	TAGACTTCTGGGTGGCCAAAGAATCA	26	
16S rRNA	16sf1F140	CGYAAGGGAAHGCTGAAA	18	Zhang and Hanner^20^
	16sf1R1524	CCGGTCTGAACTCAGATCACGTAG	24

**Table 2:** Composition of PCR master mix for COI and 16S rRNA.

Components	Volume/reaction (μL)
Template (200 ng/µL)	0.5
Buffer (10 ×)	1.25
Forward primer	0.5
Reverse primer	0.5
dNTPs (10 mM)	0.25
Taq polymerase (5 units/µL)	0.15
D/W	9.35
The total volume was 12.5 µL

**Table 3:** Thermal regime for cytochrome C oxidase I (COI) gene.

Steps	Condition		Cycles
	Temperature (°C)	Time	
Initial denaturation	94	5 min	1 cycle
Denaturation	94	30 s	35 cycles
Annealing	54	30 s	
Extension	72	45 s	
Final extension	72	7 min	1 cycle
Soak	4	Forever	–

**Table 4:** Thermal regime for 16S rRNA gene.

Steps	Condition		Cycles
	Temperature (°C)	Time	
Initial denaturation	95	5 min	1 cycle
Denaturation	95	40 s	35 cycles
Annealing	53	60 s	
Extension	72	45 s	
Final extension	72	7 min	1 cycle
Soak	4	Forever	–

#### Immunocytochemistry

Morphology of the OCF cells was checked by immunocytochemistry using monoclonal antibodies against Vimentin (V6630-CLONE 9 Sigma) and Cytokeratin (C2931-Clone C-11 Sigma). Briefly, the cells were grown on cover slips upto 90% confluency in a 12 well tissue culture plates (Nunc). The cells were washed with PBS and fixed in 4% p-formaldhyde (PFA) and again washed twice with PBS. The cells were permeabilized with 0.1% Triton X-100. To prevent the unspecific binding of the antibodies, blocking was done with PBS containing 5% sheep serum for 40 min at 37 °C. The blocking solution was removed and 100 μL of anti-Vimentin (1:40 dilution) along with anti-pan cytokeratin (1:200 dilution) was added and incubated for overnight at 4 °C. The reaction was carried out in duplicate wells. The cover slip was washed with PBS to remove any unbound antibodies and treated with 100 μL of FITC-labelled anti-mouse IgG (1:300 dilution). The cover slip containing stained cells was washed again with PBS and mounted using 50% glycerol (in PBS) and observed under fluorescent microscope^52^.

### Chromosome analysis

OCF cells at passage 19th were used for chromosome analysis. The cells were inoculated in a 25 cm^2^ culture flask and incubated for 24 h. Subsequently, 10 µL of 0.5% Colchicine (Sigma-Aldrich, St. Louis, MO) was added to the cells and incubated for 2 h in BOD incubator at 28 °C. Cells were removed from the flask by trypsinization and the cell pellet was collected by centrifugation at 1500×*g* for 5 min. The pellet was gently resuspended in 0.56% KCl and incubated for 20 min at room temperature for swelling. The hypotonic cell pellet was collected by centrifugation at 1500×*g* for 5 min. The hypotonic action was stopped by gradually adding 1 mL freshly prepared chilled Carnoy’s fixative (Methanol: Glacial Acetic acid in 3:1) and mixed gently and the cell pellet was collected by centrifuging at 1500×*g* for 5 min. The above step of fixing was repeated 2–3 times by fresh fixative till clear transparent cell suspension was obtained. A small quantity of cell suspension was taken in a pasture pipette and dropped onto grease, from a height of 1–1.5 feet on a pre-cleaned glass slide and stained with 4–5% Giemsa (pH 6.8) for 30 min. Then, slide was washed with double distilled water thoroughly. Photomicrographs of metaphase spreads were taken under the oil immersion objective (100x).

### Transfection of OCF cell line with pmaxGFP vector

The OCF cell line with 80–90% confluency at 26th passage was trypsinized from the flask and then seeded at different densities in a six-well plate, each containing 1 mL media with 10% FBS and incubated at 28 °C overnight. The culture medium was aspirated completely and gently rinsed with PBS and 1 mL of fresh serum-free media (SFM) added followed by adding 250 µL of Transfection mixture (Lipofectamine 3000 Reagent, Invitrogen) drop-wise to each well-containing cell and mixed gently. The transfected cells were incubated at 28 °C overnight in BOD incubator. The next day, 1 mL of media containing 10% serum was added to each well and incubated for 48 h at 28 °C and then observed for the expression of GFP under an inverted fluorescence microscope. The efficiency of transfection was determined by the percentage of the fluorescence protein-positive cells to the number of viable cells 48 h after the start of transfection.

### Viral susceptibility

The cell line with 80% confluent monolayers of OCF cells at the 30th subculture was selected for the virus susceptibility test. Nervous necrosis virus (NNV) infecting seabass, *Lates calcarifer* was used to test virus susceptibility of the OCF cell line. NNV inoculum was derived from the SSN cell line. The preparation of the virus for inoculation was performed and 50% tissue culture infective dose (TCID_50_) was estimated as described by Reed and Muench^21^. A negative control was included and had cells treated with a homogenate from healthy tissues and showed no NNV-associated CPE. The OCF cells were infected with NNV at a multiplicity of infection (MOI) of approximately 0.1 (formation of one pfu in 1000 cells seeded), calculated by the ratio of plaque-forming units (pfu) of virus used for infection to the number of cells seeded. The initial TCID_50_ titre of the inoculum was calculated and found to be 2.1 log TCID_50_/mL. The virus inoculum (0.2 mL) applied to the cells cultured in L-15 media with 0.2% FBS in the T-25 cell culture flask, and the culture was incubated for 12 h at 28 °C for absorption. Replaced the old medium with fresh medium containing 10% FBS and incubated at 28 °C for ten days. The cells were observed daily for any cytopathic effect (CPE) caused by the virus under an inverted microscope. The virus replication efficiency was determined using 1 mL of cultured fluid which was harvested at 3, 5, 7 and 9 days of virus inoculation^21^. The presence of the virus in the cells was confirmed through a two-step reverse-transcriptase PCR specific to NNV using primers, BNV-UF1 & UR1, and BNV-UF2 & UR2^22^. The details of primers, master mix perpetrations, and thermal regime in conventional PCR for the confirmation of NNV are mentioned in Tables 5,6 and 7.

**Table 5:** Primers used for conventional PCR for the confirmation of NNV.

Steps	Primer name	Primer sequence (5′-3′)	Length	Reference
Step 1	BNV-UF1	CAACTGACARCGAYCACACCTTCG	24	Gomez et al*.*^22^
	BNV-UR1	CGDGGYTGCKSRTCRGARTARTA	23	
Step 2	BNV-UF2	THCAAGCRACTCGYGGTGC	19	
	BNV-UR2	TGCCARTAVACRGCMCGKTCVACRTC	26	

The expected amplified product size from step 1 and step 2 was 570 bp and 420 bp.

**Table 6:** Composition of PCR master mix for both step 1 and step 2 of NNV.

Components	Volume/reaction (μL)
Template (100 ng/µL)	1
Buffer (10 ×)	2.5
Forward primer	0.5
Reverse primer	0.5
DNTPs (10 mM)	0.5
Taq polymerase (5 units/µL)	0.25
NFW	19.75
The total reaction volume was 25 µL

**Table 7:** Thermal regime for step 1 and step 2 of nested PCR for NNV.

Steps	Conditions		Cycles
	Temperature (°C)	Time	
Initial denaturation	94	3 min	1 cycle
Denaturation	94	30 s	35 cycles
Annealing	57	30 s	
Extension	72	60 s	
Final extension	72	10 min	1 cycle
Soak	4	Forever	–

$$Multiplicity\, of\, infection \left(moi\right)=\frac{Plaque\, forming\, units\, (pfu)\, of \,virus\, used\, for \,infection}{Number \,of \,cells}$$

### Short-term cryopreservation

Shor-term cryopreservation of the OCF cell line was carried out in − 80 °C ultra-freezer, and their stability was assessed according to Freshney^23^. OCF cells of 23rd passage at densities of 4 × 10^6^ cells/mL were harvested and concentrated by centrifugation. The cell suspensions were carefully resuspended in a freezing medium embrace of L-15 medium with 10% serum and 10% dimethyl sulphoxide (DMSO). Aliquots of 1 mL were dispensed into 2 mL of sterile cryovials. The cryovials were kept at 4 °C for 2 h followed by − 20 °C for 1 h, and then cryovials were kept at − 80 °C for one month. The recovery of frozen cells after one-month of post-storage was performed by thawing at 37 °C in a water bath. The cell viability was determined by staining with trypan blue, and the total viable cells were enumerated using a hemocytometer.

## Results

### Primary and subculture of OCF cells

**Figure 1:**
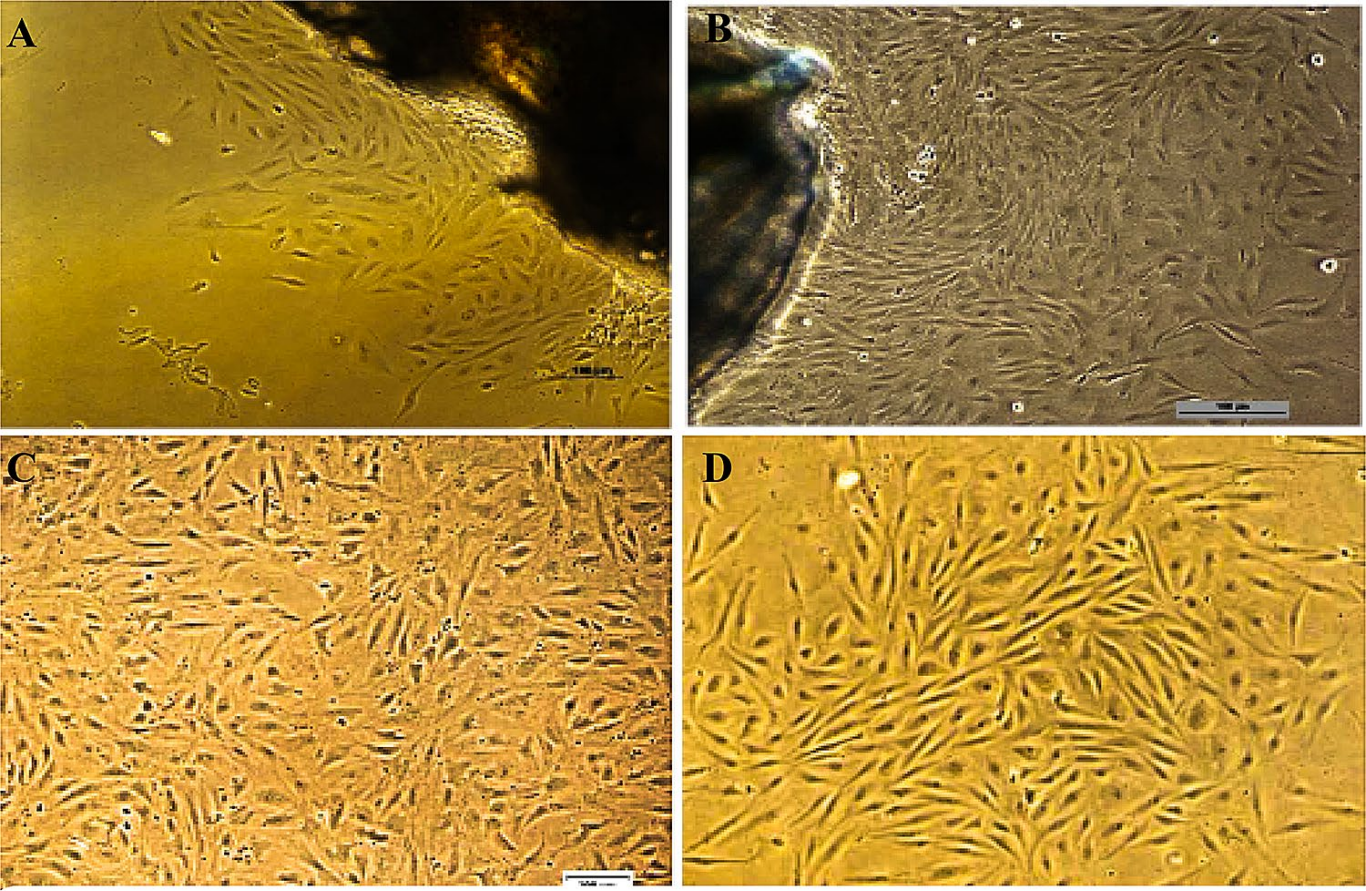
Phase contrast photomicrographs of OCF cell line. (**A**) Cells showing radiation (10 ×). (**B**) Confluent monolayer of cells around the explants (10 ×). (**C**) Cells at 10th passage (10 ×). (**D**) Cells at 34th passage (10 ×).

In the primary culture, the caudal fin explants of *A. ocellaris* were found to be properly affixed after 20 h of explant preparation and cells start radiating after 72 h of explant preparation. During the first 10 subcultures, a mix of 50% each of the fresh and spent L-15 medium with 20% FBS was utilized for 4 days of interval. In succeeding subcultures, cells were passaged using a fresh medium of L-15 with 15% FBS at 4 days of interval. OCF cell line has been subcultured or passaged and maintained up to 34 passages (Fig. 1).

### Growth studies

#### Serum concentration(s)

**Figure 2:**
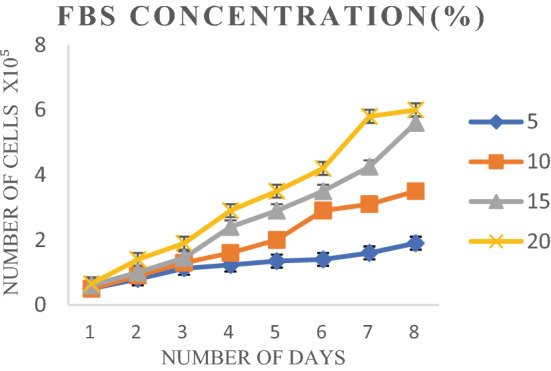
Growth of cell line at different FBS concentrations.

Cell growth was observed at different concentrations of FBS, i.e., 5, 10, 15, and 20% to find out optimum FBS concentration. OCF cells revealed poor growth at 5% of FBS concentration whereas comparatively better growth was observed at 15% (Table S1, Fig S1), but maximum growth of the cells was observed with 20% FBS concentrations (Fig. 2).

#### Salt concentration

**Figure 3:**
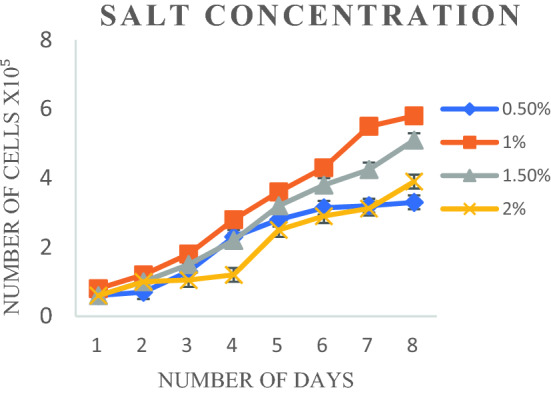
Growth of cell line at different salt concentration (0.2 M NaCl).

Cells did not survive beyond 1 or 2 days during initial subcultures. Optimization of salt concentration in the medium was indeed essential since the cells were derived from a marine fish. To estimate the optimum salt concentration to support consistent cell growth, 0.2 M sodium chloride was supplemented with L-15 medium containing 20% serum at different salt concentrations, i.e., 0.5%, 1%, 1.5%, and 2% (Table S2, Fig S2). The cells were found to grow well at 1–1.5% NaCl with maximum growth recorded at 1% (Fig. 3).

#### Temperature

**Figure 4:**
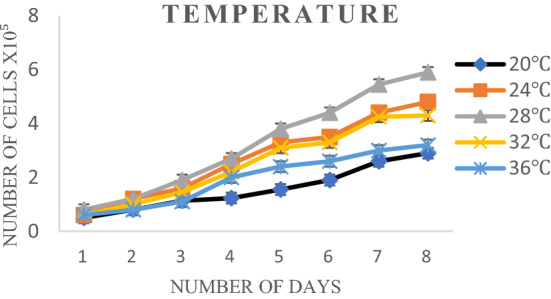
Growth of cell line at different temperatures (°C).

Cells were incubated at different temperatures i.e., 20, 24, 28, and 32 °C to find out the optimum temperature to support consistent cell growth. The growth of cells increased as the incubation temperature increased from 24 to 28 °C (Table S3, Fig S3), but the maximum growth was recorded at 28 °C (Fig. 4). No significant growth was observed at 20 and 32 °C (Error bar represents the standard deviation (SD) (n = 3)).

### Plating efficiency and cell doubling time

The maximum plating efficiency of OCF cells at 16th passage was found to be 2% when the cells were seeded at a density of 1 × 10^3^ cells per well in a 6-well plate. The estimated cell doubling time of the OCF cell line at the 25th passage was 40 h.

### Authentication of cell line using molecular markers

**Figure 5:**
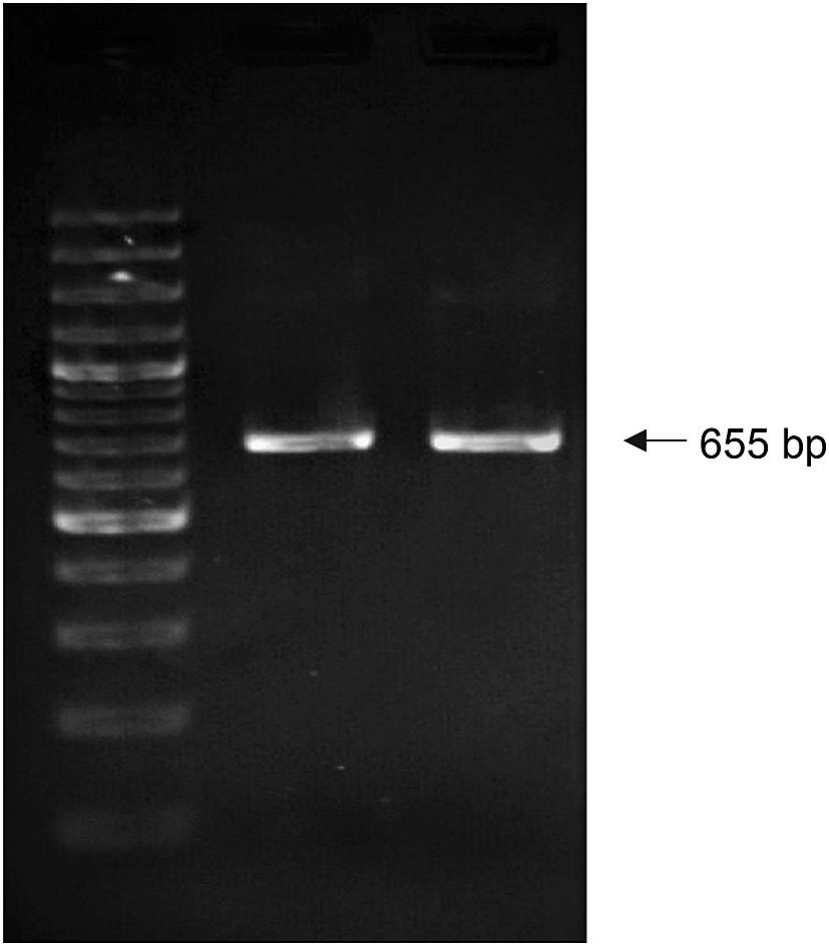
PCR Amplification of 655 bp fragment of *A. ocellaris* genome using oligonucleotide primers from the conserved portions of COI region. Lane 1—generuler express 100 bp DNA ladder (Fermentas) Lane 2—positive control, Lane 3—OCF COI.

**Figure 6:**
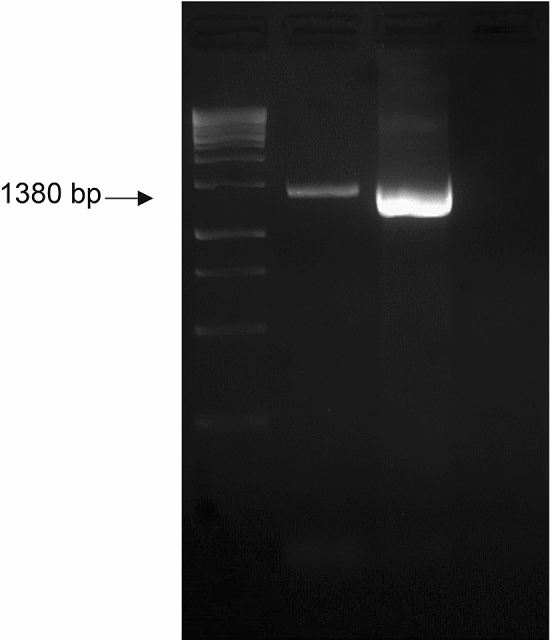
PCR Amplification of 1380 bp fragment of *A. ocellaris* genome using oligonucleotide primers from the conserved portions of 16S rRNA region. Lane 1—generuler express 1 kb DNA ladder (Fermentas) Lane 2—positive control; Lane 3—OCF 16S rRNA; Lane 4—negative control.

DNA barcode generated by PCR amplification of COI and 16S rRNA genes of the OCF cell line yielded 655 bp (Fig. 5) and 1380 bp (Fig. 6) respectively. The alignment of COI and 16S rRNA gene sequences obtained from the cell lines with the known gene sequences of *A. ocellaris* revealed a 99–100% similarity (Table 8). Thus, mitochondrial genes such as 16S rRNA and COI gene sequence derived from the cell lines authenticated the species of origin of the cell line.

**Table 8:** GenBank accession numbers of the COI and 16S rRNA genes of *A. ocellaris*.

Cell line	Mitochondrial gene	GenBank accession no.
OCF	COI	MH049232.1
	16S rRNA	AB980197.1

### Immunocytochemistry

**Figure 7:**
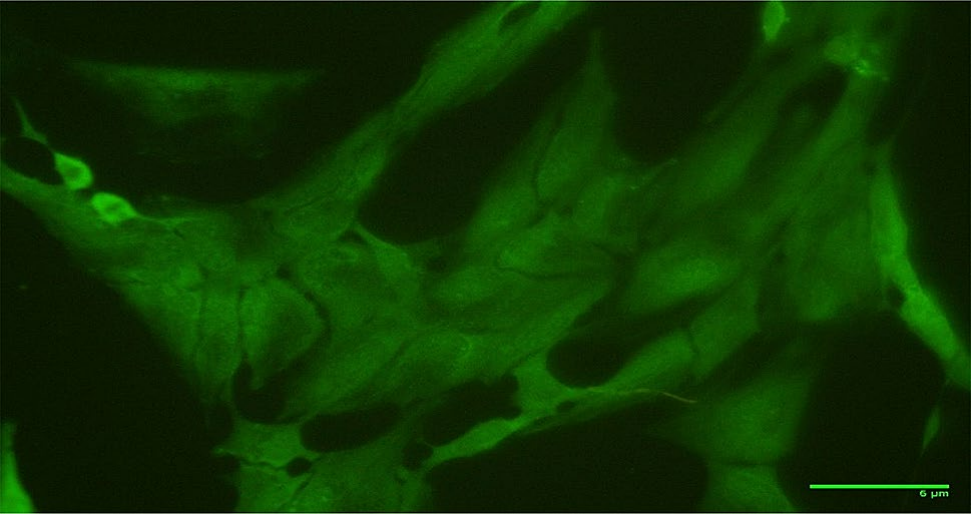
Morphological confirmation of OCF cell line by Vimentin-FITC expression (400 ×).

The expression of Vimentin-FITC in OCF cell line was observed which confirmed the fibroblastic morphology of the cell line (Fig. 7).

### Chromosome analysis

**Figure 8:**
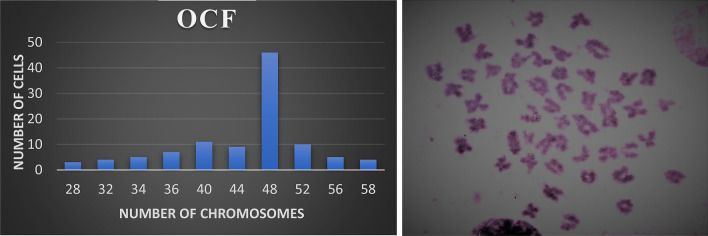
Chromosome frequency distribution and cellular chromosomes arrested in metaphase of OCF cells at 19th passage.

The chromosome counts of 104 metaphase plates revealed that the diploid number of chromosomes in the OCF cell line at 19th passage ranged from 28 to 58 (Table S4, Fig S4) with a modal value of 48 (Fig. 8).

### Transfection efficiency

**Figure 9:**
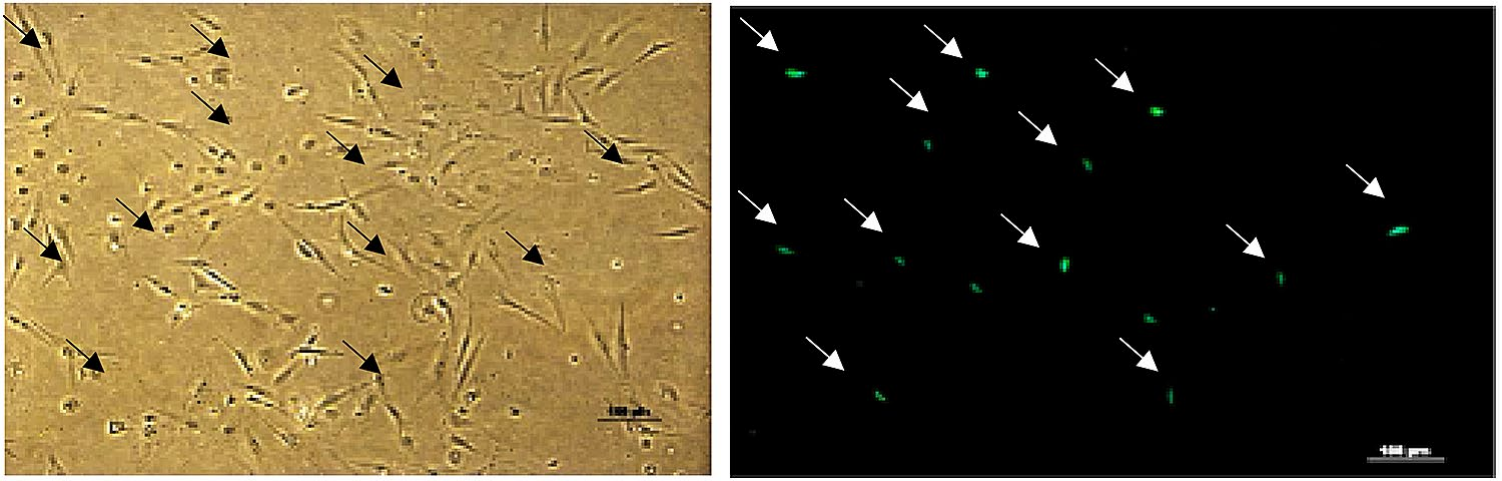
Green fluorescent protein expression of cell line transfected with pmaxGFP (10 ×).

The OCF cells at 26th passages were successfully transfected with pmaxGFP plasmid Lipofectamine 3000 Reagent from Invitrogen. The expression of pmaxGFP in the OCF cells was detected after 48 h of transfection by the observation of a clear green fluorescent signal under a fluorescent microscope (Fig. 9). The transfection efficiency of OCF cells was estimated at 7%.

### Viral susceptibility

**Figure 10:**
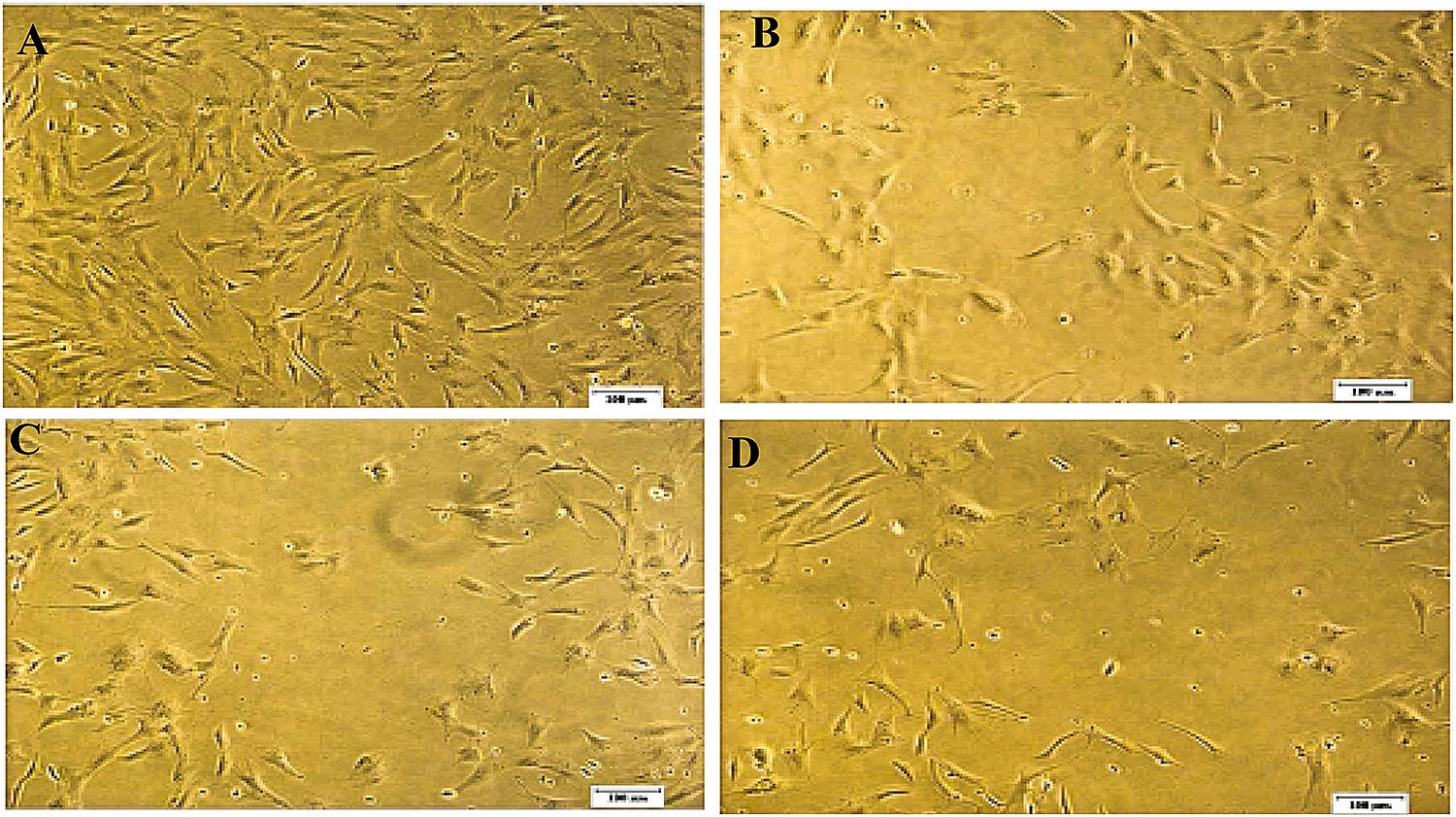
Susceptibility of OCF cells at the 30th passage to Betanodavirus. (**A**) Confluent uninfected OCF cells (10 ×). (**B**) Extensive CPE with multiple vacuolation in OCF cells infected with NNV after 3 days of post infection (10 ×). (**C**) Disintegration of more than 50% of monolayer observed after 9 days of post infection (10 ×). (**D**) Disintegration of more than 50% of monolayer observed after 13 days of post infection (10 ×).

The susceptibility of OCF cells at the 30th passage to Nervous necrosis virus (NNV) was evaluated by the observation of the cytopathic effect (CPE). The advanced CPE was observed at 24 and 48 h of post-infection. Initially, the occurrence of a lot of small foci in the monolayer with cell aggregations and lysis was observed. The virus titre (TCID_50_) was calculated and it found to be significantly increased from 2.1 log TCID_50_/mL to 3.8 log TCID_50_/mL at 9 days of post infection. As the development of CPE progressed, additional adjoining cells became detached and granular until the entire cell sheet was eventually lysed and affected completely after 7 days of post-infection (Fig. 10A–D). The monolayer of the OCF cell line completely disintegrated after 4 days of post-infection. The infection in OCF cells with NNV was further confirmed by Nested PCR.

### Confirmation of NNV by nested PCR

**Figure 11:**
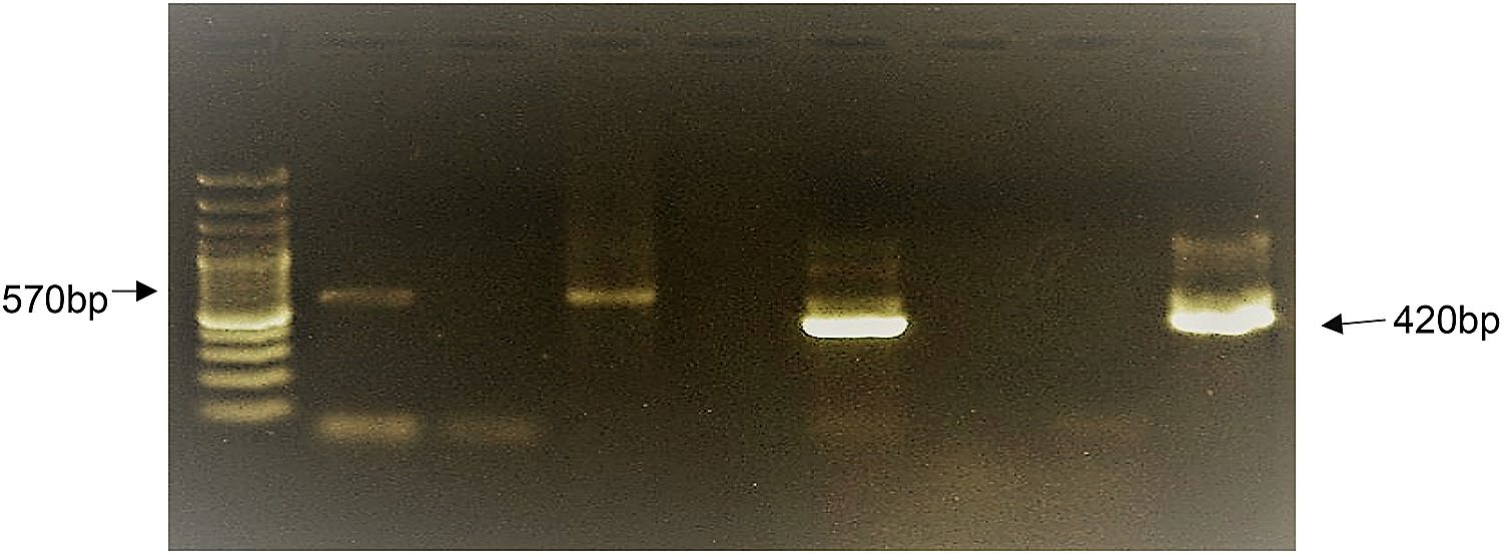
PCR Amplification by nested PCR for the confirmation of NNV genome using specific primers. Lane 1—generuler express 100 bp DNA ladder (Fermentas); Lane 2—NNV for first step; Lane 3—negative control; Lane 4—positive control; Lane 6—NNV for second step; Lane 7—negative control; Lane 8—nested negative; Lane 9—positive control for second step.

The amplification of the NNV genome was performed by nested PCR using specific gene primers. The cDNA of infected cells amplified with NNV primers exhibited positive results in the expected amplified size of 570 and 420 bp (Fig. 11) in the first and second steps, respectively. The results confirmed the susceptibility of OCF cells to NNV.

### Short-term cryopreservation

The cryopreserved OCF cells at 23rd passage following dimethyl sulfoxide (DMSO) freezing procedure at − 80 °C showed a successful revival of the cells after thawing. The average estimated revival percentage was 70–75% of the initial cell population. The revived cells recovered well and grew to confluency within 7 days.

## Discussion

The present study was carried out to develop and characterize a new cell line from *A. ocellaris.* The primary culture developed from caudal fin by explant technique has several advantages over the trypsinization method in terms of speed, ease, and maintenance of cell interactions and the avoidance of enzymatic digestion which can damage the cell surface^24^. Patkaew et al. were the first to report on the cell culture system developed from the ocellaris clownfish. Their study describes only a simple explant method from the vertebra up to 4th passage, using RPMI‐1640 supplemented with 20% FBS and they studied its growth characteristics^25^. Nanda et al. studied the comparison of the explant method and trypsinization method and found better attachment of cells by using the explant technique compared to the trypsinization method in *Cirrhinus mrigala*^26^. The radiation of cells from the primary cultures consisted of a heterogeneous population, containing both epithelial and fibroblast cells. Such type of heterogeneous group of cells during the establishment of primary cultures was reported by many researchers^27–29^. The process for the development of cell lines was standardized to promote cell growth as cells did not survive beyond 1 or 2 days during initial subcultures. After the optimization of salt concentration in the medium, it was possible to subculture the cells, which ultimately resulted in stable cell lines. Adjustment of salt concentration to maintain osmolality was quite crucial for the development of cell lines. The osmolality of commercial L-15 varies between 300 and 340 mOsmkg^−1^ and standard preparations of L-15/ex fell into this range, with a mean of 326 mOsmkg^−1^ T 9 (n = 8)^30^. In the process of subsequent passaging, the cells of caudal fin were successfully subcultured up to 34 passages and it was designated as OCF. Domination of fibroblast cells over epithelial cells was observed in the OCF cell line from the 10th passage onwards under an inverted microscope. The predominance of fibroblast cells over epithelial cells in cell cultures from fish was reported in previous studies^31–35^.

The maximum growth of the OCF cell line was observed in the L-15 medium with 20% FBS, followed by a gradual decrease in the FBS concentration to 15% for subsequent passage after the 34th subculture. About 15–20% FBS was required during the initial stages of development^15,28,36–38^. The osmolality of marine fish blood is generally around 250 to 400 mOsmkg^–1^, and the marine fish cell line survives well within this range^39,40^. Osmolality can be adjusted with addition or dilution of salts. During the initial subculture, the cells grew very fast at the beginning of day one, afterward, there was a drastic slow growth and finally, total cell death occurred. To overcome the problem, the culture media L-15 was supplemented with different concentrations of 0.2 M sodium chloride solution, and it was found that OCF cells grew well and formed a complete monolayer at 2 mM NaCl. The fish cell line can grow in a wide range of temperatures from 24 to 32 °C^34,41–43^. In the present study, the most suitable temperature for optimum growth and proliferation of the developed OCF cell line was revealed to be 28 °C which showed conformity with other marine fish cell cultures^44^. Two cell lines from carp have been growing well at 37 °C^45^.

The maximum plating efficiency observed for the OCF cell line at 16th passage was 2% when seeded at 1000 cells. The high proliferation of cells and plating efficiency observed in the cell lines is expressive of a transformed characteristic or genotypic change^46^. Goswami et al. reported 64% of plating efficiency seeded at 1000 cells per flask^43^. The gene sequence of both COI and 16S rRNA obtained from the OCF cells revealed the similarity of 99–100% with the known sequences of *A. ocellaris* submitted in NCBI, Genbank Database. Hebert^47^ has demonstrated the usage of the mitochondrial gene, COI as a universal barcode, designated as—DNA barcoding for the genetic recognition of animal life and it has also been used to specify the species and to study relationships between the species^48^. Other alternatives to COI, such as the 16S rRNA gene sequence can also be used to confirm the origin of fin and muscle cell lines^49^. Chromosomal analysis indicates that the OCF cell line at the 19th subculture possessed a diploid chromosomal number of 2n = 48 which was alike to the modal chromosomal number of *A. ocellaris*^50^. The immunocytochemistry based on the Vimentin-FITC expression revealed the fibroblastic morphology of the OCF cell line which showed conformity with the previous studies for the confirmation of fibroblast morphology^34,51–54^. However, additional markers like CauVim and CauK8-IIS, CauK49-IE and CauK50-IE, the tight junction protein, zonula occludens-1 (ZO-1), would be useful to support the confirmation of the fibroblast morphology of the cell line^55,56^.

In the current study, the OCF cell of the 26th subculture was successfully transfected with Lipofectamine 3000 reagent to determine the efficiency of transfection from the commercially accessible transfection reagents**.** The estimated transfection efficiency from the OCF cell line was low, i.e., 7%, which can be comparable to 10% efficiency reported in the PSCF cell line^34^. However, Zhou et al. showed a transfection efficiency of 2% in a CSTF cell line established from *Acipenser sinensis*^57^*.* The OCF cell line could be utilized for the production of recombinant proteins and gene expression studies using improved transfection methods e.g. nucleofection other than lipofectamine.

Isolation and propagation of recently emerging nodavirus^16^ were reported in clown fish^58,59^. The OCF cell line of the 30th subculture exhibited high susceptibility to nervous necrosis virus (NNV), and the susceptibility of the virus to the OCF cell line was confirmed through VNN-specific nested PCR. The increasing rate of virus titre was found to be similar with Parameswaran et al.^60^. The study validated the potential of the OCF cell line as a robust in vitro tool for the isolation and identification of NNV for studying the pathogenesis in *A. ocellaris.*

The OCF cell line was successfully cryopreserved by a slow freezing procedure using dimethyl sulphoxide (DMSO) at − 80 °C with 70–75% of cell revival of the initial cell population. Cryopreservation of fin cells derived from glass catfish by 15% DMSO slow-freezing method facilitates the recovery rate of 95% of cells^8^. Patkaew et al. cryopreserved the vertebra cell line of ocellaris clownfish at 5th passage in liquid nitrogen and obtained a viability rate of 80%^25^.

## Conclusion

The OCF cell line was successfully developed from the caudal fin of *A. ocellaris*. The process for the development of cell lines was standardized to promote cell growth as cells did not survive beyond 1 or 2 days during initial subcultures. After the optimization of salt concentration in the medium, it was possible to subculture the cells, which ultimately resulted in stable cell line. The cell line was characterized for species authentication, chromosomal analysis, growth potential at ifferent temperatures, FBS concentrations, and salt concentrations. The revival ability of cells was evaluated by short-term cryopreservation. In vitro transfection efficiency of the cell line was assessed by the expression of green fluorescent protein (GFP). The OCF cell line was tested for virus susceptibility. The study revealed that the OCF cell line would be a useful tool for virological studies. In short, the developed cell line from *A. ocellaris* would be a useful tool for in vitro research and conservation genetics of this important ornamental fish species.

## Supplementary information

Supplementary Information.
