# Supplementary material for: Characterization of a new cell line from ornamental fish Amphiprion ocellaris (Cuvier, 1830) and its susceptibility to nervous necrosis virus
Source: Sci Rep. 2020 Nov 18;10:20051. doi: 10.1038/s41598-020-76807-7 (PMC7676255; doi:10.1038/s41598-020-76807-7)
Supplement: Supplementary file 1 — Supplementary Information. [file 41598_2020_76807_MOESM1_ESM.docx]

**Characterization of a newly Developed Cell Line OCF from the Caudal Fin of Marine Ornamental Fish *Amphiprion ocellaris* (Cuvier, 1830) for Virological Studies**

Yashwanth B.S**^1^**, Mukunda Goswami**^1*^**, K.V Rajendran**^2^**, Dimpal Thakuria**^3^**, and Aparna Chaudhari**^1^**

**Table S2: Growth of cells at different Temperature with respect to time (in days)**

**Table S1: Growth of cells at different concentrations of FBS with respect to time (in days)**

| **Temperature** | **20℃** | **24℃** | **28℃** | **32℃** | **36℃** |
| --- | --- | --- | --- | --- | --- |
| 1 | 0.5 | 0.6 | 0.8 | 0.6 | 0.6 |
| 2 | 0.8 | 1.2 | 1.2 | 1 | 0.8 |
| 3 | 1.13 | 1.6 | 1.9 | 1.45 | 1.1 |
| 4 | 1.23 | 2.5 | 2.7 | 2.2 | 2 |
| 5 | 1.55 | 3.3 | 3.8 | 3.1 | 2.4 |
| 6 | 1.9 | 3.5 | 4.4 | 3.3 | 2.6 |
| 7 | 2.6 | 4.4 | 5.45 | 4.25 | 3 |
| 8 | 2.9 | 4.8 | 5.9 | 4.3 | 3.2 |

| **FBS conc. (no. of days)** | **5%** | **10%** | **15%** | **20%** |
| --- | --- | --- | --- | --- |
| 1 | 0.5 | 0.5 | 0.6 | 0.65 |
| 2 | 0.8 | 0.9 | 1 | 1.4 |
| 3 | 1.13 | 1.3 | 1.45 | 1.9 |
| 4 | 1.23 | 1.6 | 2.4 | 2.9 |
| 5 | 1.35 | 2 | 2.9 | 3.5 |
| 6 | 1.4 | 2.9 | 3.5 | 4.2 |
| 7 | 1.6 | 3.1 | 4.25 | 5.8 |
| 8 | 1.9 | 3.5 | 5.6 | 6 |

| **Salt conc. (no. of days)** | **0.50%** | **1%** | **1.50%** | **2%** |
| --- | --- | --- | --- | --- |
| 1 | 0.6 | 0.8 | 0.6 | 0.6 |
| 2 | 0.7 | 1.2 | 1 | 1 |
| 3 | 1.3 | 1.8 | 1.5 | 1.05 |
| 4 | 2.3 | 2.8 | 2.2 | 1.2 |
| 5 | 2.8 | 3.6 | 3.2 | 2.5 |
| 6 | 3.14 | 4.3 | 3.8 | 2.9 |
| 7 | 3.2 | 5.5 | 4.25 | 3.12 |
| 8 | 3.3 | 5.8 | 5.1 | 3.9 |

**Table S3: Growth of cells at Salt concentrations with respect to time (in days)**

**[Error bar represents the standard deviation (SD) (n=3)]**

**Fig S2: Growth of cell line at different salt concentration (0.2 M NaCl)**

**Fig S1: Growth of cell line at different FBS concentration**

**Fig S3: Growth of cell line at different temperatures (⁰C)**

**Chromosome Preparation**

**Table S4: Chromosome frequency distribution with respect to number of microslides prepared**

| **Number of Chromosomes** | **Number of cells/slides** |
| --- | --- |
| 28 | 3 |
| 32 | 4 |
| 34 | 5 |
| 36 | 7 |
| 40 | 11 |
| 44 | 9 |
| 48 | 46 |
| 52 | 10 |
| 56 | 5 |
| 58 | 4 |

**Fig S4: Chromosome frequency distribution and cellular chromosomes arrested in metaphase of OCF cells at 19^th^ passage.**
